# Strengthening Cause of Death Statistics in Selected Districts of 3 States in India: Protocol for an Uncontrolled, Before-After, Mixed Method Study

**DOI:** 10.2196/51493

**Published:** 2024-12-20

**Authors:** Ashoo Grover, Saritha Nair, Saurabh Sharma, Shefali Gupta, Suyesh Shrivastava, Pushpendra Singh, Srikanta Kanungo, Senthanro Ovung, Charan Singh, Abdul Mabood Khan, Sandeep Sharma, Subrata Kumar Palo, Tapas Chakma, Anjali Bajaj

**Affiliations:** 1 Indian Council of Medical Research New Delhi India; 2 Indian Council of Medical Research-National Institute for Research in Digital Health and Data Science New Delhi India; 3 Indian Council of Medical Research-National Institute of Research in Tribal Health Jabalpur India; 4 Indian Council of Medical Research-Regional Medical Research Centre Bhubaneswar India; 5 Indian Council of Medical Research-National JALMA Institute for Leprosy & Other Mycobacterial Diseases Agra India; 6 Community Health Center, Haripur Kangra India

**Keywords:** cause of death, Medical Certification of Cause of Death, capacity building, Civil Registration and Vital Statistics, training

## Abstract

**Background:**

Mortality statistics are vital for health policy development, epidemiological research, and health care service planning. A robust surveillance system is essential for obtaining vital information such as cause of death (CoD) information.

**Objective:**

This study aims to develop a comprehensive model to strengthen the CoD information in the selected study sites. The specific objectives are (1) to identify the best practices and challenges in the functioning of the Civil Registration and Vital Statistics (CRVS) system with respect to mortality statistics and CoD information; (2) to develop and implement interventions to strengthen the CoD information; (3) to evaluate the quality improvement of the Medical Certification of Cause of Death (MCCD); and (4) to improve the CoD information at the population level through verbal autopsy for noninstitutional deaths in the selected study sites.

**Methods:**

An uncontrolled, before-after, mixed method study will be conducted in 3 blocks located in the districts of 3 states (Madhya Pradesh, Uttar Pradesh, and Odisha) in India. A baseline assessment to identify the best practices and challenges in the functioning of the CRVS system, along with a quality assessment of the MCCD, will be conducted. An intervention informed by existing literature and the baseline assessment will be developed and implemented in the study sites. The major components of intervention will include a Training of Trainers workshop, orientation of stakeholders in the functioning of the CRVS system, training of physicians and medical officers in the MCCD, and training of community health workers in World Health Organization Verbal Autopsy 2022 instrument. Postintervention evaluation will be carried out to assess the impact made by the intervention on the availability and quality improvement of CoD information in the selected study sites. The outcome will be measured in terms of the quality improvement of the MCCD and the availability of CoD information at population level through verbal autopsy in the selected study sites.

**Results:**

The project has been funded, and regulatory approval has been obtained from the Institutional Ethics Committee. The data collection process began in May 2023. The duration of the study will be for 24 months.

**Conclusions:**

Our study is expected to provide a valuable contribution toward strengthening CoD information, which could be helpful for policy making and further research. The intervention model will be developed in collaboration with the existing functionaries of the health and CRVS systems in the selected study sites that are engaged in reporting and recording CoD information; this will ensure sustainability and provide lessons for upscaling, with the aim to improve the reporting of CoD information in the country.

**International Registered Report Identifier (IRRID):**

DERR1-10.2196/51493

## Introduction

The Civil Registration and Vital Statistics (CRVS) system is the key source of vital statistics [1-4]. It maintains data regarding registered births and deaths, provides death and birth certifications, and publishes vital statistics, which also include cause of death (CoD) information [4]. Additionally, censuses, sample surveys, household surveys, and facility-based service statistics are also used to estimate death rates [5,6]. Information about mortality statistics and ascertaining the CoD are significant components for the formulation of health policies, epidemiological research, health care service planning, and addressing public health emergencies [6-8]. Many of the current health goals—for instance, reducing child and maternal mortality and reducing noncommunicable disease deaths—are aligned with United Nations Sustainable Development Goals [1,7,9,10]. Having effective mortality surveillance systems to monitor the progress toward achieving Sustainable Development Goals is essential [5,8,11].

To strengthen the CRVS system, some efforts have been made at the global and regional levels, such as the *World Health Organization (WHO) CRVS Strategic Implementation Plan 2021-2025* [10], Data for Health initiative by Bloomberg Philanthropies [12,13], and the Africa Programme on Accelerated Improvement of CRVS [12]. In India, the Registration of Births and Death Act was enacted in 1969, which makes it mandatory for citizens of India to register all births, deaths, and stillbirths [14]. According to the annual report on vital statistics of India based on the civil registration system published by the Office of Registrar General of India, the overall level of registration of deaths in India has increased from 7,641,076 in 2019 to 8,115,882 in 2020 [15]. However, only 22.5% of the total registered deaths are medically certified CoD [15,16]. There is a wide variation among the states of India, with Goa (100%) recording the highest number of medically certified deaths and Bihar (3.4%) having the lowest rate [16]. The Medical Certification of Cause of Death (MCCD) scheme is mainly done in a small number of health care facilities concentrated in urban areas of India [16-19].

Further, the majority of teaching hospitals delegate the responsibility for death certificate completion to resident physicians, with only a small number receiving formal training [20-22]. Medical students and resident physicians regularly make mistakes when filling out death certificates, leading to incompleteness and poor quality of CoD information [20,21]. Most of academic medical centers do not provide specific training in the MCCD [18,22,23].

To address the gap in the reporting the mortality statistics, the Sample Registration System under the Registrar General of India regularly conducts verbal autopsy (VA) for noninstitutional deaths. Some studies have shown that VA is a reliable source for noninstitutional deaths [12,24]. There is a significant prospect for VA to complement the MCCD, because in the absence of routine, medically certified CoD information for all deaths, VA has the potential to provide CoD information at the community level [25-29].

Our study presents a novel approach to strengthening CoD information by developing a comprehensive model that includes a baseline assessment of the functioning of the CRVS system, which will enable us to develop appropriate interventions or provide recommendations to have a systematic and efficient approach to improve CoD information [12,30,31]. Additionally, improving the skills and capacities of physicians for certifying CoD [12,18,32-34], along with providing VA training to community health workers and sensitizing key stakeholders to VA, could be helpful in improving the quality and availability of CoD information. Our approach could also be useful for other settings where CRVS systems face challenges in the death registration process and in obtaining CoD information [4,28]. The study aims to develop a comprehensive model to strengthen CoD information in the selected study sites. The objectives are (1) to identify the challenges and best practices in the functioning of the CRVS system with respect to mortality statistics and CoD information; (2) to develop and implement interventions to strengthen CoD information; (3) to evaluate the quality improvement of the MCCD; and (4) to improve CoD information at the population level through VA for noninstitutional deaths in the selected study sites.

## Methods

### Study Design

An uncontrolled, before-after, mixed method study will be conducted to identify the best practices and challenges in the existing CRVS system with respect to mortality statistics and the generation of CoD information. The intervention will consist of training in the MCCD and VA for health personnel, in addition to designing appropriate interventions or providing recommendations. The outcome will be assessed by evaluating the quality of the MCCD (Form 4; the physician fills out the form to certify the CoD in case of institutional deaths), as well as in the availability of CoD information at the population level through VA for noninstitutional deaths in selected study sites.

### Study Setting

The study will be conducted in 3 blocks located in the districts of 3 states (Odisha, Madhya Pradesh, and Uttar Pradesh) in India. As shown in Table 1, these states were selected based on the low number of medically certified deaths compared to registered deaths and the presence of a Model Rural Health Research Unit (MRHRU) [16]. MRHRUs are a Government of India initiative that was established with the goal of improving the quality of health care services in rural India. The MRHRUs serve as a hub for translational research that can be extrapolated all throughout the Indian public health care system. They also serve as an interface between researchers, health care providers, and communities.

**Table 1:** Comparison of total registered deaths with medically certified deaths in the selected states^a^.

State	Total registered deaths (in 100,000), n	Total medically certified deaths (in 100,000), n
Odisha	362,982	59,296
Madhya Pradesh	524,454	35,105
Uttar Pradesh	873,419	109,688

^a^Data sourced from the *Report on Medical Certification of Cause of Death* [16].

### Study Duration

As depicted in Table 2, the duration of the study will be for 24 months.

**Table 2:** Gantt chart for the activities planned in the study.

Activities planned	First year					Second year			
	Q1	Q2	Q3	Q4	Q1		Q2	Q3	Q4
Preparatory phase (desk review, recruitment of staff, and orientation of staff with the study objectives)	✓	✓							
Pilot-testing of the baseline assessment tool, training of staff, key informant interviews with stakeholders, and development of the implementation plan		✓	✓						
Implementation of interventions, including a Training of Trainers workshop, orientation of stakeholders on the functioning of CRVS^a^, training of physicians in MCCD^b^, training of community health workers, and sensitization of key stakeholders				✓	✓		✓		
Monitoring, process evaluation, and endline assessment							✓	✓	
Analysis, discussion with stakeholders, and dissemination									✓

^a^CRVS: Civil Registration and Vital Statistics.

^b^MCCD: Medical Certification of Cause of Death.

### Sampling

This will be a pilot study primarily focusing on feasibility, proposing a model, and obtaining primary information with respect to the quality and availability of the CoD information in the selected study sites.

#### Qualitative Data

Keeping in mind the availability of time and resources, a total of 10-15 key informant interviews (KIIs) will be conducted at the district, block, and *panchayat* (village) levels from each study site to gather the relevant information related to mortality statistics and CoD information (Multimedia Appendices 1-2). A list of persons in the study blocks who are knowledgeable and engaged in any way on issues pertaining to recording and generating mortality statistics (registration of deaths and generation of CoD information) will be populated by the research team in consultation with the district health officials. Two separate lists will be generated, one for the key personnel from the community and the another for those who are in official positions and are engaged in the CRVS system. These lists will be used for the selection of the key informants.

#### Quantitative Data

Considering the feasibility, 10% of the MCCD forms (Form 4) will be randomly selected from all institutional deaths, excluding medicolegal cases, from the selected public health care facility in the study sites for quality assessment; similarly, if available, the quality of VA forms will also be assessed [35-38]. As per the MCCD rapid assessment tool that will be used to assess the quality of the MCCD forms [39], a total of 330-300 MCCD forms (approximately 110-100 MCCD forms each study site) will be chosen for 1 year (April 2022 to March 2023) from the public health care facility.

### Data Collection

**Figure 1:**
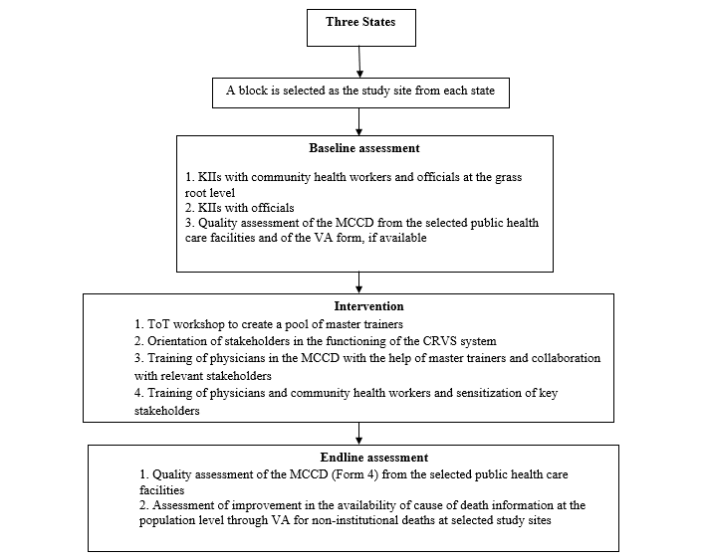
Flow chart of the methods. CRVS: Civil Registration and Vital Statistics; KII: key informant interview; MCCD: Medical Certification of Cause of Death; ToT: Training of Trainers; VA: verbal autopsy.

As per the methods shown in Figure 1, the baseline assessment will include the following.

#### Qualitative Assessment

Following selection from the lists prepared by the research team, an appointment will be sought and those consenting for the interviews will be contacted as per the time given. A total of 10-15 KIIs will be conducted for each study site at the district, block, and *panchayat* levels. Probes will comprise major domains such as practice on death registration, suggestions to improve the death registration, CoD information, and perceptions on public awareness. These will be used to gather the information with respect to mortality statistics and CoD information.

#### Quantitative Assessment

After obtaining permission from the local health authorities, MCCD forms will be accessed from the selected public health care facility for quality assessment. As per the MCCD rapid assessment tool that will be used to assess the quality of the MCCD forms [39], a total of 330-300 MCCD forms (approximately 110-100 MCCD forms each study site) will be chosen for 1 year (April 2022 to March 2023) from the public health care facility. Physicians trained during the Training of Trainers (ToT) workshop will assess the MCCD forms for quality. Quality assessment for VA will also be conducted, depending on its availability at the selected study site.

### Intervention

Intervention activities will include the following strategies.

#### Baseline Assessment

Baseline assessment will help us to identify the challenges and best practices in the CRVS system with respect to mortality statistics and CoD information, as well as to inform us for the development of appropriate interventions or to provide recommendations. A series of stakeholder orientation meetings will be conducted to inform the baseline results and seek the active engagement of the stakeholders in strengthening the reporting of CoD for noninstitutional deaths.

#### Training of Physicians on the MCCD

As suggested through existing literature, the training of physicians is crucial for improving the quality and availability of CoD information.

Trainers will be identified at the institutes, MRHRUs, and district and government medical colleges. A ToT workshop will be planned. The agenda for the ToT workshop (physicians and medical officers) on improving medical certification of CoD will be adapted from existing training materials [40-44] and will include topics such as (1) introduction to death reporting forms and its importance (Form 2, Form 3, Form 4, and Form 4a); (2) guidelines, procedure, and steps for the MCCD through specific reference to diseases; (3) common errors in the MCCD; (4) a brief session on International Classification of Diseases coding; and (5) medical certification of CoD—quality assessment. Each training for physicians and medical officers will last 2 days and will be planned according to the days convenient for them in small batches. The training will be conducted in collaboration with relevant stakeholders and master trainers at each study site.

#### Training for Health Workers for Conducting VA Using the WHO VA 2022

In parallel, frontline workers such as auxiliary nurse midwifes, community health officers, and physicians will be trained in the WHO VA 2022 instrument for noninstitutional deaths [25,45-47]. Additionally, the sensitization of key stakeholders will be done to make them aware of the significance of VA in improving CoD information.

### Outcome Measures

We will use the MCCD rapid assessment tool to evaluate the quality of MCCD forms, for which we have referred to some studies to gain insights into the postintervention assessment [36,39,42,48]. Table 3 provides the variables that will be assessed.

**Table 3:** Key variables that will be assessed after the intervention.

Category	Variables
General details about the deceased	Age Gender
Death certificate details	Name of the certifier Trained certifier
Completeness and accuracy	Multiple cause of death mentioned Time interval missing from onset to death Use of abbreviations Illegible handwriting Clinically incorrect sequence of events Incorrect underlying cause

### Endline Assessment

#### Improvement in the Quality of the MCCD

Improvement in the overall quality of the MCCD (Form 4) will be assessed for 6 months from the same public health care facility selected for baseline assessment in each study site. The quality assessment will be done using the MCCD rapid assessment tool [35-37].

#### Improvement in the Quality of CoD Information

Likewise, improvement in the availability of CoD information at the population level through VA for noninstitutional deaths will be assessed.

### Data Management

#### Qualitative Analysis

Thematic analysis will be conducted to analyze the information collected through KIIs. Themes, categories, and codes will be synthesized to analyze the data.

#### Quantitative Analysis

All the categorical variables, such as demographic details of the deceased, the name of certifier, time interval missing from onset to death, use of abbreviations, illegible handwriting, etc, will be summarized as frequencies and percentages. The chi-square test will be used to evaluate the improvement in the quality of the MCCD (based on the MCCD rapid assessment tool) before and after the intervention.

### Ethical Considerations

The Institutional Ethics Committee of the Indian Council of Medical Research–National Institute for Research in Digital Health and Data Science (erstwhile National Institute of Medical Statistics) has approved the study (NIMS/IEC/03/2022). Participant information sheets (English, Hindi and Oriya) will be developed to communicate the objectives of the study to the stakeholders. Written informed consent will be obtained from the stakeholders before their inclusion in the study. The data collection will start in the first year.

## Results

The project has been funded. The data collection process began in May 2023, which will be followed by training of the physicians and community health workers in the MCCD and WHO VA 2022 instrument. The endline assessment is expected to be completed in the initial months of the second year of study duration. The duration of the study will be for 24 months.

## Discussion

An uncontrolled, before-after, mixed method study will be conducted in the blocks of 3 states in India. These states were selected based on the low number of medically certified deaths compared to total registered deaths and the presence of MRHRUs. This study aims to develop a comprehensive model to strengthen the CoD information in the selected study sites. The intervention includes a ToT workshop, orientation of stakeholders in the functioning of the CRVS system, training of physicians and medical officers in the MCCD, and training of community health workers in the WHO VA 2022 instrument. Postintervention assessment will be conducted to evaluate the impact made by the intervention on the availability and improvement in the quality of CoD information in the selected study sites. The outcome measures will be (1) improvement in the quality of the MCCD forms and (2) improvement in the availability of CoD information at the population level through VA in the selected study sites.

Various strategies have been implemented to improve the CoD information, such as research studies, standard guidelines, training in the MCCD, and use of the VA method. The *WHO CRVS Strategic Implementation Plan 2021-2025* is an initiative to help member states improve the availability of mortality data and certification processes for CoD [10]. The Africa Programme on Accelerated Improvement of CRVS is a regional program to strengthen the CRVS system in the African continent [12]. These initiatives necessitate further efforts to improve the quality and availability of CoD information.

This study focuses on developing appropriate interventions or providing recommendations to address the challenges and understand best practices in the existing CRVS system. KIIs will be conducted to attain this objective. A similar method was used in a study conducted in Zambia by Yokobori et al [31] to assess the CRVS system’s performance. This involved conducting a questionnaire based interviews in health facilities.

As a part of intervention, training and capacity building for physicians and medical officers in the MCCD will be conducted at the selected study sites. Myer and Farquhar [20] conducted a similar study, where a 75-minute seminar was conducted as an educational intervention for internal medicine residents on completeness of death certification. Another study was conducted to understand the knowledge, behavior, and attitude of house officers and general practitioners regarding death certification and to ascertain the possibility of developing appropriate intervention in future [22].

Furthermore, this study recognize the importance of involving other stakeholders, such as police officials, *panchayat* leaders, community health workers, etc, that play a crucial in reporting and recording mortality data, especially noninstitutional deaths. To improve the quality and availability of CoD information at the population level through VA, this study will conduct training on the WHO VA 2022 instrument for community health workers along with sensitizing key stakeholders in VA. A study conducted in Thailand by Polprasert et al [26] demonstrated the significance of VA in improving the CoD information, aligning with one of the objectives of this study.

The strengths of the study are as follows: (1) it is one-of-a-kind study where a comprehensive model will be developed to improve the quality and availability of CoD information; (2) it will be a multicenter study that could help us to have a better understanding of the best practices and challenges in the reporting and recording of CoD information; (3) it will involve key stakeholders other than health care professionals in the process of improving CoD information; and (4) the MCCD rapid assessment tool will be used to evaluate the quality of the MCCD before and after the intervention. However, this study may also face some limitations. It does not address the training of mortality coders to accurately code the CoD information provided on the MCCD.

This study is expected to provide a valuable contribution toward strengthening CoD information, which could be helpful for policy making and further research. The intervention model will be developed in collaboration with the existing functionaries of the health and CRVS systems in the selected study sites that are engaged in reporting and recording CoD information; this will ensure sustainability and provide lessons for upscaling, with the aim to improve the reporting of CoD information in the country.

## Appendix

Multimedia Appendix 1 Key informant interview questionnaire for officials.

Multimedia Appendix 2 Key informant interview questionnaire for frontline workers.

## Abbreviations

CoD: cause of death
CRVS: Civil Registration and Vital Statistics
KII: key informant interview
MCCD: Medical Certification of Cause of Death
MRHRU: Model Rural Health Research Unit
ToT: Training of Trainers
VA: verbal autopsy
WHO: World Health Organization
