# Supplementary material for: Strengthening Cause of Death Statistics in Selected Districts of 3 States in India: Protocol for an Uncontrolled, Before-After, Mixed Method Study
Source: JMIR Res Protoc. 2024 Dec 20;13:e51493. doi: 10.2196/51493 (PMC11699485; doi:10.2196/51493)
Supplement: Multimedia Appendix 1 [file resprot_v13i1e51493_app1.doc]

**Key Informant Interview Guide-1**

*Guide for Key Informant Interview with Officials*

1. **Background characteristics**

- Age
- Sex
- Residence
- Education
- Type of health facility (currently working)
- Working experience (years in service)
- Duration of living in current residence

1. **Death registration in the CRVS**

- Are you aware of laws or legislations which are applied for registration of deaths? What are they? What is your opinion on the practice of these here (in you locality)?
  1. *Presence of legislation that states death registration is compulsory*
  2. *Presence of regulations to report all death events to vital statistics system within a given time*
- What is the role of medical officer/health assistant in death registration?
- Please inform on the death registration process in the block (stakeholders/personnel involved, explore on registration of deaths at health facility (or) at home, capacities of health system, challenges), if yes, what steps have been taken to overcome them?

1. How are death-recorded/ reported from lower level to higher level (from local to regional to central office)? Registration process in the stipulated time period as mentioned by CRVS?
2. Availability of facilities to carry out necessary functions (e.g. offices, forms, papers, books, telephones, computers)
3. Any trainings received to carry out necessary functions (to whom, how often) of death registration? If yes, when the last training happen and what was the content? Were you part of any of these

- Are you aware of any meetings/ committees for checking of completeness of registration of deaths in this facility? If yes, how? By whom? Frequency?

1. When did the most recent evaluation to assess completeness of death registration in this facility happen?

- Are you aware of laws or legislations which are applied for recording of cause of deaths information? What are they? What is your opinion on the practice of these here (in your locality)?
  1. Presence of legislation that states cause of deaths information is compulsory
  2. Who can certify the cause of death? Presence of legislation that states death has to be certified by cause?
- What is the role of medical officer/health assistant in recording/ reviewing cause of deaths information?
- How death certification is performed within and outside hospitals? How is it different within and outside hospital?

1. Use of standard international form of medical certificate of cause of death for reporting
2. Use of verbal autopsy to determine the cause of death when medical certification of cause of death is rare

- How do you make sure to obtain quality cause-of-death data in this area?

1. Training of doctors for certifying cause of death
2. **Perceptions on public awareness and practice on death registration and need for reporting of cause of death information)**

- Explore on awareness,
- knowledge,
- attitude and
- practice of people towards registering deaths in the block
- Are any activities undertaken for creating awareness registration of vital event? Who does it?
- Do communities perceive any benefits of registering deaths? If yes, what are the perceived benefits? How about registration of births?

1. **(ONLY TO CRVS OFFICIALS)**

Death registration data access, dissemination and use

- Availability of the annual numbers of deaths in your area? Are they disaggregated by sex and age?
- Is there a delay between the reference year and the time when detailed statistics on cause of death are made available to the public?
- How are these data used for policy and program purposes? Suggestions to improve the death registration and CoD information in the area
- What are the challenges that you or your staff face in recording and registering deaths, issuing certificates and reporting of cause of deaths?
- What are the suggestions for further improving these in this district?

**(Only for CBHI officials):**

- What are the challenges you have faced regarding the Implementation of the trainings?
- What is the frequency of training?
- Are physicians willing to join the sessions? If not, what are the reasons you have perceived regarding it?
- How much adherence to the sessions have you seen during the training?
- How many trainers are required for each session?
- How is the date and time decided by you for the trainings?
