# Supplementary material for: Strengthening Cause of Death Statistics in Selected Districts of 3 States in India: Protocol for an Uncontrolled, Before-After, Mixed Method Study
Source: JMIR Res Protoc. 2024 Dec 20;13:e51493. doi: 10.2196/51493 (PMC11699485; doi:10.2196/51493)
Supplement: Multimedia Appendix 2 [file resprot_v13i1e51493_app2.doc]

**Key Informant Interview -II**

*Guide for Key Informant Interviews Health Workers/ officials at the grass root level*

1. **Background characteristics**
2. Age
3. Sex
4. Residence
5. Position
6. Name of ward or village or block
7. Education
8. Working experience (duration)
9. Duration of living in current residence (years)
10. **Death recording, reporting** **of cause of death**
11. Who are the other stakeholders involved within this area for death registration process? What are their roles and responsibilities? Please explain the process of death occurring in hospitals/ home.
12. If the person is involved: what is your role in death recording and reporting?
13. Please elaborate on the process of registration in the block/ district. (documentation required, automated process) What are the challenges that you face in death reporting and registration in your area?
14. Do death certificates have cause of death information? How is it recorded? Kindly elaborate on the mechanisms to record the cause of death of a person, if the death has occurred in a hospital/community?
15. Are you aware of verbal autopsy, if yes, how did you come to know about it? Are these conducted in the health facility if yes, who does it?
16. Have you received any training on death registration/ collecting information on causes of death? If yes, when was the training received and what was the content of the training? Do you feel there is any need for refresher training? If yes, what should be the content?
17. Are review meetings on death registration held at the block/district/Gram Panchayat level? If yes, what is the frequency of such meetings? What issues are generally discussed? Is there any feedback mechanism?
18. **Perceptions on public awareness and practice on death registration and reporting of cause of death information**
19. What do you think about public awareness and participation in death registration?
20. The success of registration of vital event to a greater extent depends on the awareness level of the communities. Were there any IEC activities undertaken by you for death registration in the community? If yes, what were those
21. Do communities perceive any benefits of registering deaths, particularly in terms of access to government services? If yes, what are the perceived benefits?
22. Are people aware about recording of cause of death information
